# Effect of Macronutrient Composition on Appetite Hormone Responses in Adolescents with Obesity

**DOI:** 10.3390/nu11020340

**Published:** 2019-02-05

**Authors:** Kay Nguo, Maxine P Bonham, Helen Truby, Elizabeth Barber, Justin Brown, Catherine E Huggins

**Affiliations:** 1 Department of Nutrition, Dietetics and Food, Monash University, Notting Hill 3168, Australia; maxine.bonham@monash.edu (M.P.B.); helen.truby@monash.edu (H.T.); elizabeth.barber@monash.edu (E.B.); kate.huggins@monash.edu (C.E.H.); 2 Monash Children’s Hospital, Monash Medical Centre, Clayton 3168, Australia; justin.brown@monashhealth.org; 3 Department of Paediatrics, Monash University, Clayton 3168, Australia

**Keywords:** obesity, adolescent, appetite, ghrelin, GLP-1

## Abstract

Gut appetite hormone responses may be influenced by meal macronutrients and obesity. The primary aim of this study was to examine in adolescents with obesity and of healthy weight the effect of a high-protein and a high-carbohydrate meal on postprandial gut appetite hormones. A postprandial cross-over study with adolescents 11–19 years old was undertaken. Participants consumed, in random order, a high 79% carbohydrate (HCHO) and a high 55% protein (HP) meal. Ghrelin, glucagon-like peptide 1 (GLP-1), peptide YY (PYY), and self-reported appetite were assessed for four hours postprandial. Total energy intake from an ad libitum lunch and remaining 24 h was assessed. Eight adolescents with obesity (OB) and 12 with healthy weight (HW) participated. Compared with HW, OB adolescents displayed a smaller ghrelin iAUC (−25,896.5 ± 7943 pg/mL/4 h vs. −60,863.5 ± 13104 pg/mL/4 h) (*p* = 0.008) with no effect of meal (*p* > 0.05). The suppression of ghrelin relative to baseline was similar between OB and HW. Ghrelin suppression was greater following the HP vs. HCHO meal (effect of meal, *p* = 0.018). Glucose and insulin response were greater following HCHO vs. HP, with responses more marked in OB (time × weight × meal interaction, *p* = 0.003 and *p* = 0.018, respectively). There were no effects of weight or macronutrient on GLP-1 or PYY, appetite or subsequent energy intake. The present study demonstrates that dietary protein can modulate postprandial ghrelin responses; however, this did not translate to subsequent changes in subjective appetite or energy intake.

## 1. Introduction

Lifestyle interventions are integral to the management of overweight and obesity [1]. Dietary protein is considered a more satiating macronutrient than carbohydrate and fat and is often promoted as an important component of diet-based weight management [2,3]. This may, in part, be due to the effects of protein on satiety signals (neuro-endocrine signals). The gastrointestinal tract has important endocrine functions designed to regulate energy intake by the secretion of a number of appetite-regulating hormones, such as the orexigenic hormone ghrelin and the anorexigenic hormones peptide YY (PYY) and glucagon-like peptide-1 (GLP-1) [4]. Manipulation of dietary macronutrients (protein, carbohydrate, and fat) may regulate the secretion of these hormones, thereby potentially acting to influence appetite and energy intake.

In adults, carbohydrate and protein have been shown to be more effective at suppressing ghrelin levels than fat [5]. Protein has been shown to promote the stimulation of GLP-1 and PYY more so than carbohydrate or fat [6], albeit inconsistently [7]. Moreover, the relationship between macronutrients and appetite hormone secretion in adults may, in part, be modulated by body weight. In adults with obesity compared with those of healthy weight, reduced postprandial PYY secretions have been observed after a high-protein meal [8], as well as reduced suppression of ghrelin after a mixed meal [9]; by contrast, no differences in PYY and ghrelin responses after meal intake have also been reported between these groups [10].

In children and adolescents, very little research on the impact of macronutrients on appetite hormone secretion has been conducted, in particular examining the effect of obesity on these endocrine responses. A recent systematic review and meta-analysis [11] identified only two studies investigating acute macronutrient–appetite hormone relationships in childhood—one study with prepubertal children (7–11 years) [12] and another study with adolescent girls (12–18 years) [13]. Moreover, according to the authors’ knowledge, no studies in young people have examined the acute postprandial response of GLP-1 to different macronutrients. Adolescence is a time of rapid growth and tremendous hormonal changes, including pubertal alterations in ghrelin [14]; hence, it may not be possible to extrapolate data from prepubertal children and adults to adolescents.

Effective weight management in adolescents can reduce disease risk in adulthood. Understanding the release of endogenous satiety hormones through modulation of diet composition may provide further insight into the development of practical nutrition strategies for adolescent obesity management. The primary aim of this study was to examine the gastrointestinal appetite hormone responses to a high-protein and a high-carbohydrate meal in adolescents with obesity compared with those of healthy weight. It was hypothesised that (1) protein compared with carbohydrate will promote a gastrointestinal hormone profile reflecting greater satiety and that (2) responses will be different between adolescents with obesity and those of healthy weight.

## 2. Materials and Methods

### 2.1. Study Design and Testing Procedures

**Figure 1:**
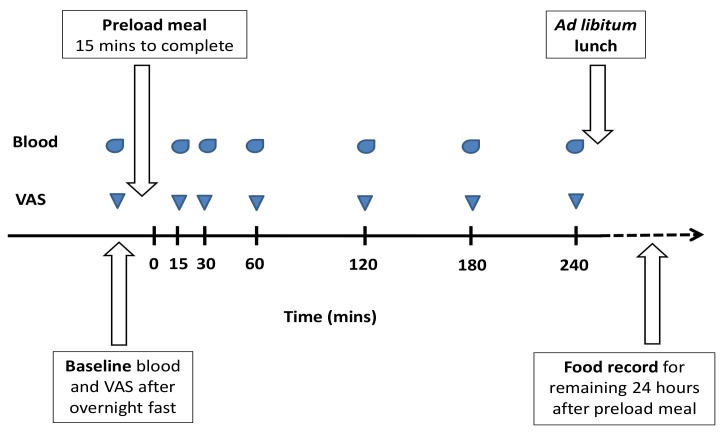
Testing day timeline. Oval shape indicates blood sampling and downwards triangle indicates hunger and fullness measures by visual analogue scales (VAS).

The design was a double-blinded cross-over study. Participants reported to the test laboratory in Melbourne (between 07:30 h and 08:00 h) on two separate occasions (with a minimum one-week washout period) after an overnight 12-h fast and 24-h avoidance of strenuous physical activity. Baseline anthropometric measures and visual analogue scales (VAS) assessing subjective hunger and fullness were completed (100 mm line with questions of “how hungry do you feel” and “how full do you feel”, with anchors at both ends of ‘not at all’ to ‘extremely’). An intravenous cannula was inserted, and baseline blood was drawn. A high-protein or high-carbohydrate liquid breakfast meal was provided to participants in a random order, and they were asked to consume this within 15 min. Blood sampling and VAS were completed at 15, 30, 60, 120, 180, and 240 min postprandial (timed from after the meal was eaten). Only water (no food) and nonstrenuous sedentary activities were permitted over the postprandial period. At the end of the four hours, an ad libitum lunch (a commercially prepared lasagne and diced fruit) was served in a quiet room without any observation. Participants were instructed to eat until ‘comfortably full’ within 30 min. Food intake was determined by weighing all food before and after consumption, after which participants went home. All food and beverages consumed for the remaining 24 h were self-recorded in a food diary. Energy intake (kJ) as determined from the weight of food consumed in the laboratory and the self-recorded food diary using Foodworks (V7.0.3016; Xyris Software, Australia). See Figure 1 for testing day timeline.

### 2.2. Test Meals

Either a high-protein (whey) (55% protein, 30% carbohydrate, 15% fat) or high-carbohydrate (maltodextrin) (79% carbohydrate, 5% protein, 16% fat) liquid meal was provided on each of the two testing mornings. The meals were matched for percentage of fat but differed in the percentage of protein and carbohydrate. The energy content of the meals was provided as 25% of each participant’s estimated energy requirements (EER). EER was estimated using basal metabolic rate (Schofield equation for children and adolescents) x activity factor [15], with activity factor estimated by the physical activity questionnaire for adolescents (PAQ-A) [16]. The ingredients of each meal consisted of milk, vanilla ice cream, and canola oil and either whey protein isolate (Ascend Sport, Melbourne, Australia) or Poly Joule™ powder for the high-protein and high-carbohydrate meals, respectively. The selection of a liquid for the test meal was made for ease of adjusting the energy content to 25% of each participant’s energy requirements as well as for keeping volume and energy density similar on each occasion. The primary purpose of this study was to examine the ‘macronutrient effect’ on outcomes rather than food form per se; hence, a liquid meal was chosen.

### 2.3. Participant Recruitment

Participants were eligible if they were aged between 11–19 years old with obesity (BMI ≥95th percentile) or of healthy weight (BMI 5–85th percentile), based on the United States-Centers for Disease Control and Prevention (US-CDC) charts. Participants were recruited via community advertising or from a paediatric weight management clinic. Exclusion criteria were: Taking medications known to alter body composition or metabolism; obesity due to secondary causes relating to genetic or endocrine related disorders; lactose intolerant or following restricted diets. Pubertal stage was assessed using the Tanner stage [17,18]. Participants provided informed assent and their parent’s or guardian’s written informed consent. This study was conducted according to the guidelines laid down in the Declaration of Helsinki, and all procedures involving human participants were approved by the Monash Health Human Research Ethics Committee (HREC) (Ref 12014B) and the Monash University HREC (Project Code CF12/1987-2012001086).

### 2.4. Anthropometry and Body Composition

Anthropometric measures were determined without shoes and in light clothing. Height was measured using a stadiometer (Holtain Ltd., Crymych, United Kingdom), weight using an electronic scale (SECA Group, Hamburg, Germany), and umbilical waist and hip circumference using a stretch-resistant tape (Figure Finder, Novel Products, Rockton, IL, USA). All measures were taken in duplicates. Body composition was estimated using bioelectrical impedance (BIA) (Bodystat QUADSCAN 4000, Bodystat Limited, Isle of Man, British Isles). Impedance (50 kHz) was used to estimate fat free mass (FFM) using the equation of Houtkooper et al. [19]: FFM = 0.61 × (Height (cm)^2^/impedance (50kHz)) + 0.25 × weight (kg) + 1.31 (Height in cm) was used to estimate FFM in those who were healthy weight. The equation derived by Haroun et al. [20]: FFM = −2.211 + 1.115 (Height (cm)^2^/ impedance (50 kHz)) was used to estimate FFM in those with obesity. Fat mass was calculated as weight minus FFM.

### 2.5. Blood Sampling and Analysis

Blood samples were collected into 8-mL BD800 tubes containing protease, esterase, and DPP-IV inhibitors (Becton, Dickinson and Company, Franklin Lakes, NJ, USA). The samples were centrifuged within 30 min of collection at 1300 rcf at 21 °C for 15 min and plasma-stored at −80 °C for batch analysis. Leptin (intra assay CV <6.0% and inter assay CV <6.0%), insulin (intra assay CV <7.0% and inter assay CV <9.0%), and total PYY (intra assay CV <9.0% and inter assay CV <14.0%) were measured using a Milliplex metabolic panel (Merck Millipore, Billerica, MA, USA). Total ghrelin (intra assay CV <4.0% and inter assay CV <9.2%) was measured using ELISA (Merck Millipore, Billerica, MA, USA) and total GLP-1 (intra assay CV <8.0% and inter assay CV <4.0%) was measured using ELISA (Epitope Diagnostics, San Diego, CA, USA). Glucose (intra assay CV <1.0%) was measured using the Indiko™ Clinical and Specialty Chemistry System (Thermo Fisher Scientific, Waltham, MA, USA). The HOMA (homeostasis model assessment) index was calculated as fasting glucose (mmol/L) × fasting insulin (µU/mL)/22.5 [21].

### 2.6. Statistical Analysis

Data were analysed using a mixed between–within-subjects analysis of variance, with meal and time as the within subject factor and weight (healthy weight or obese) as the between subject factor. Each model was rerun with the addition of the covariates age, gender, and pubertal stage, and results were reported only if there was a difference. The level of significance was accepted at *p* < 0.05. Data was expressed as mean ± SEM unless otherwise stated. Analyses was performed using Statistical Package for Social Sciences (SPSS) version 21.0 (SPSS Inc., Chicago, IL, USA).

## 3. Results

### 3.1. Participants’ Baseline Characteristics

Twelve adolescents with obesity and fifteen of healthy weight consented to participate. *n* = 4 obese and *n* = 3 healthy weight discontinued due to difficulties with cannulation; therefore, *n* = 8 obese and *n* = 12 healthy weight were the included in this final analysis. Baseline characteristics are presented in Table 1. A number of total PYY results were below a detectable level (<13.7 pg/mL), resulting in a PYY sample size of *n* = 5 adolescents with obesity and *n* = 5 of healthy weight adolescents.

### 3.2. Postprandial Hormone Responses

The incremental (above or below baseline) area under the curve (iAUC) for ghrelin, glucose, insulin, GLP-1, and PYY is shown in Table 2. Total concentration of ghrelin was higher in the healthy weight group compared with those with obesity (*p* = 0.008 for effect of group). No effect of meal or weight group were observed for GLP-1 and PYY concentrations (*p* > 0.05). Total insulin and glucose concentrations were greater in those with obesity compared with those of healthy weight (*p* < 0.05 for effect of group), and a greater response was seen overall after the high-carbohydrate compared with after the high-protein meal (*p* < 0.05 for effect of meal). A difference in insulin response between meal type was no longer apparent when the covariates gender and pubertal stage were accounted for (*p* = 0.472 for effect of meal).

**Figure 2:**
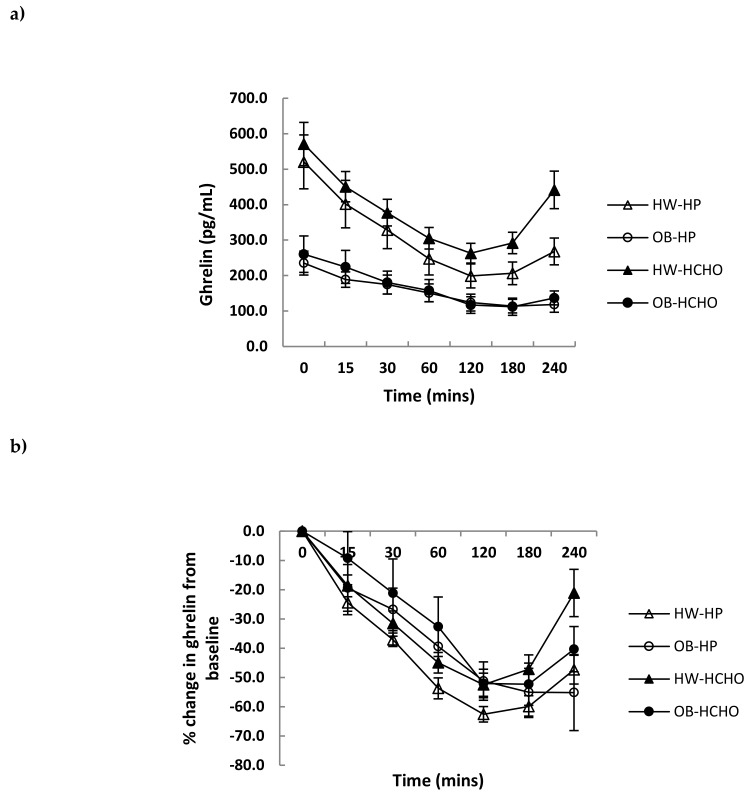
Changes in plasma ghrelin concentrations over time after a high-protein (HP) and high-carbohydrate meal (HCHO) in adolescents with obesity (OB) *n* = 8 and of healthy weight (HW) *n* = 12. (**a**) ghrelin: Effect of time (*p* = 0.001) and time x weight interaction (*p* = 0.044); (**b**) ghrelin (percentage change from baseline): Effect of time (*p* < 0.001) and meal (*p* = 0.018). Data analysed using a mixed between–within-subjects analysis of variance. Data are presented as means (SEM).

**Figure 3:**
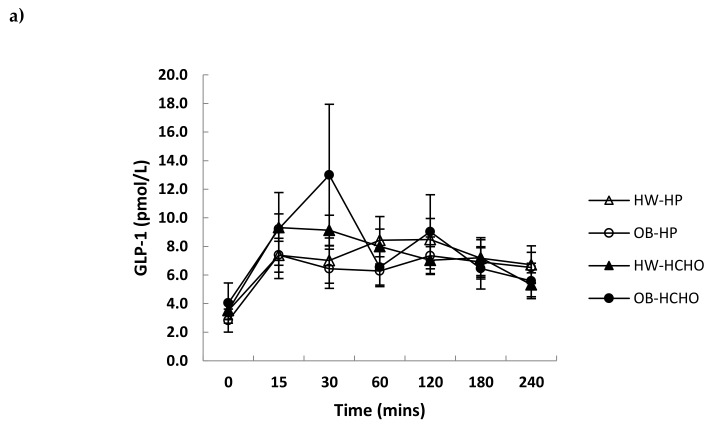

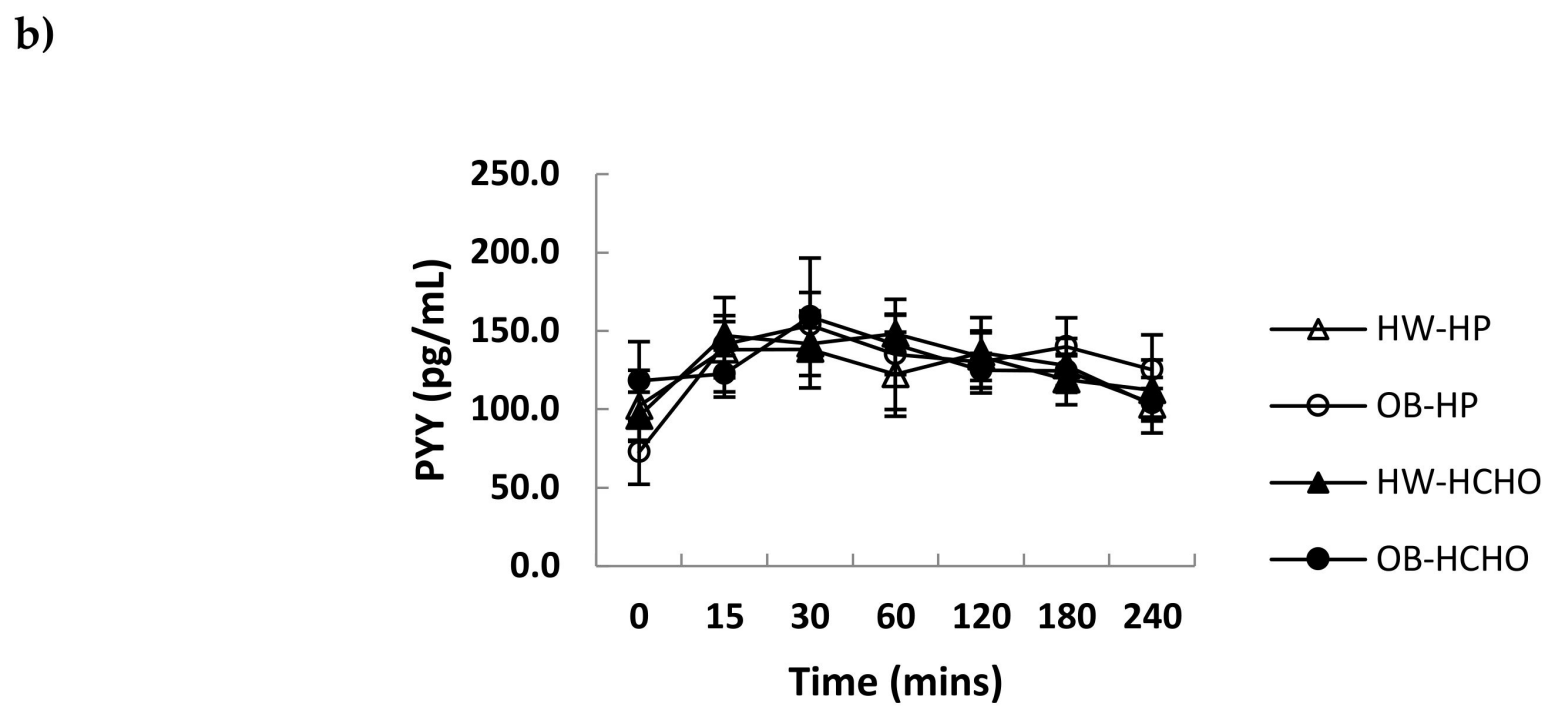
Changes in plasma GLP-1 and PYY concentrations over time after a high-protein (HP) and high-carbohydrate meal (HCHO) in adolescents with obesity (OB) and of healthy weight (HW). (**a**) GLP-1: Effect of time (*p* < 0.001) (OB *n* = 8, HW *n* = 12); (**b**) PYY (peptide YY): No effect of time or meal, or interaction effects with weight (OB *n* = 5, HW *n* = 5) (*p* > 0.05 for all values). Data analysed using a mixed between–within-subjects analysis of variance. Data are presented as means (SEM).

**Figure 4:**
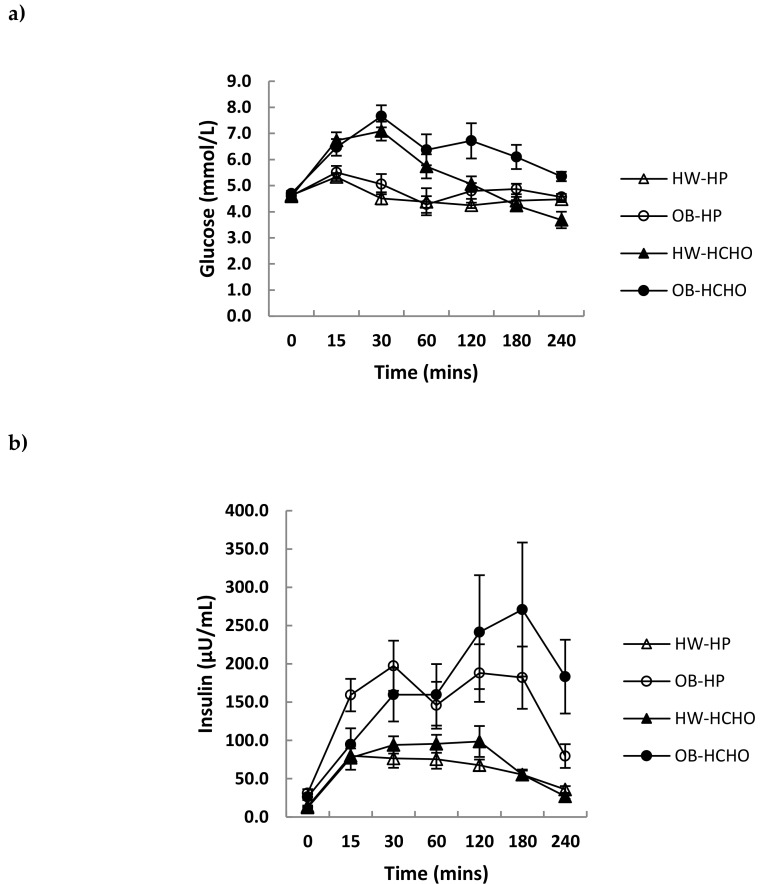
Changes in plasma glucose and insulin concentrations over time after a high-protein (HP) and high-carbohydrate meal (HCHO) in adolescents with obesity (OB) *n* = 8 and of healthy weight (HW) *n* = 12. (**a**) glucose: Time × meal × weight interaction (*p* = 0.003), (**b**) insulin: Time × meal × weight interaction (*p* = 0.018). Data analysed using a mixed between–within-subjects analysis of variance. Data are presented as means (SEM).

Over the postprandial time course, the absolute decline in plasma ghrelin was greater for adolescents who were healthy weight compared with those with obesity, which was independent of meal type (Figure 2a; *p* = 0.044 for time x weight interaction, *p* = 0.051 for effect of meal). The relative decline after adjusting for different fasting levels (expressed as a percent change from baseline) was not different between adolescents of a healthy weight and those with obesity (Figure 2b; *p* = 0.081 for time x weight interaction). A main effect of meal was observed, with the high-protein meal having a significantly greater effect on ghrelin suppression compared with the high-carbohydrate meal (Figure 2b; *p* = 0.018 for effect of meal). Postprandial changes in GLP-1 and PYY were similar (*p* > 0.05) after each test meal for both weight status groups (Figure 3a,b). Postprandial glucose and insulin were greater following the carbohydrate compared with the high-protein meal, and this was more marked in adolescents with obesity compared with those of healthy weight (time × weight × meal interaction, *p* = 0.003 and *p* = 0.018, respectively) (Figure 4a,b).

### 3.3. Self-Reported Appetite and Energy Intake

**Figure 5:**
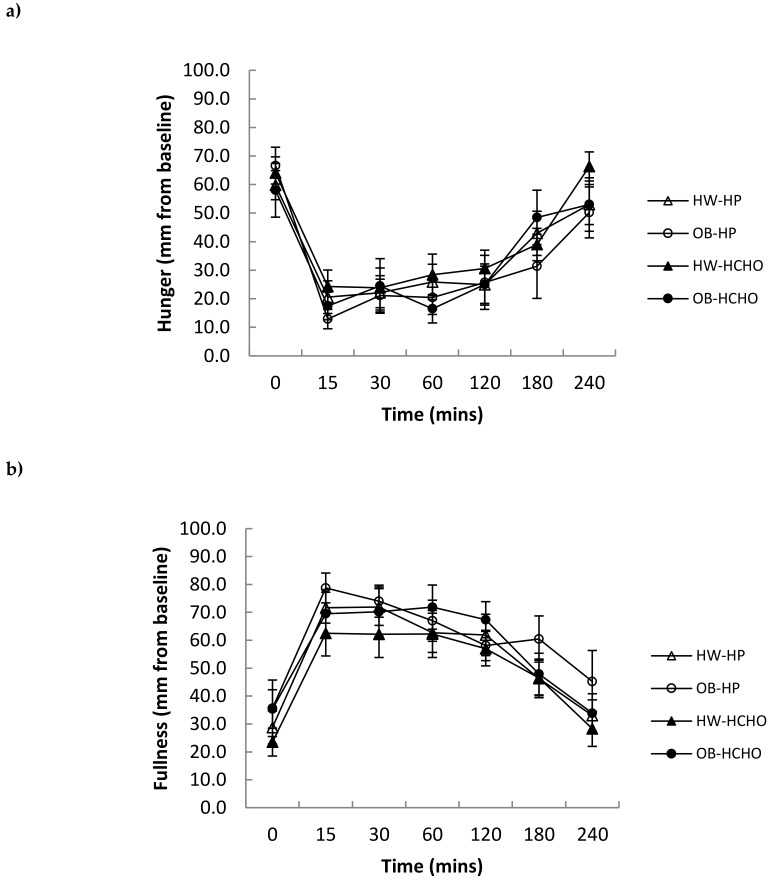
Changes in (**a**) hunger and (**b**) fullness scores over time, after a high-protein (HP) and high-carbohydrate meal (HCHO) in adolescents with obesity (OB) *n* = 8 and of healthy weight (HW) *n* = 12. There was a significant main effect of time for hunger (*p* < 0.001) and fullness (*p* = 0.001). Data analysed using a mixed between–within-subjects analysis of variance. Data are presented as means (SEM).

Self-reported hunger and fullness were not different after both meal types between adolescents with obesity and those of healthy weight (Figure 5a,b). Energy intake (kJ) at the ad libitum lunch and subsequent 24 h were also not different following the high-protein and high-carbohydrate meals in either group (Table 3).

## 4. Discussion

This study examined the acute appetite hormone responses to meals in adolescents with obesity compared with those of healthy weight. The overall finding was that meals differing in macronutrient composition can affect postprandial ghrelin as well as glucose and insulin responses. The absolute decline in ghrelin concentration following meal intake was much smaller in adolescents with obesity compared with those of healthy weight. When this was expressed as a percentage change from baseline, there was no difference between the groups; however, an effect of protein on ghrelin suppression was found overall (i.e., when both adolescents of healthy weight and those with obesity were combined). Postprandial GLP-1 and PYY responses were similar after both of the test meals.

In agreement with our hypothesis and previous literature [13,22], a smaller absolute decline in postprandial ghrelin from baseline in adolescents with obesity compared with healthy weight was observed. This smaller fall may potentially be consequential to lower baseline levels, suggesting that ghrelin secretion may already be partially suppressed in individuals with obesity, thus limiting the same magnitude of decrease compared with higher levels in those of healthy weight [23]. Lower basal ghrelin has been speculated as being part of an adaptive response to higher energy reserves and over nutrition [13,24]. When the decrease in ghrelin in this study was expressed as a percentage of baseline values, however, no significant difference existed between the two groups. The similar magnitude of ghrelin suppression between groups suggests that adolescents with obesity may not necessarily display impaired response in gut–brain appetite signals. This is congruent with Stock et al. [25], who demonstrated that although adolescents with obesity had lower basal ghrelin levels, they exhibited the same percentage change after meal intake as those of healthy weight.

No differences in postprandial GLP-1 response were seen between adolescents with obesity and those of healthy weight after meal intake, consistent with Lomenick et al. [26]. This is in contrast to other studies showing an attenuated GLP-1 response in children with obesity compared to those of healthy weight, albeit following mixed meals rather than macronutrient specific [27,28]. The heterogeneity of the studies, such as varying age groups, test meal composition and form, differing laboratory methods, including stabilisation of blood at collection, duration of the postprandial period, as well as the time from when postprandial sampling commenced, are all factors that could contribute to conflicting results. An attenuated postprandial PYY response in young people with obesity compared with that in those of healthy weight has been shown from a recent meta-analysis of a small number of studies [11]. This was not observed in this study, possibly confounded by the small sample size.

Consistent with our hypothesis, a high-protein compared with a high-carbohydrate meal promoted an attenuated rebound of the relative change in ghrelin over time. This effect of protein on the suppression of ghrelin is consistent with another study in younger children [21] and those in adults [5,29], although no effects of protein on ghrelin suppression have also been shown [7]. A high-carbohydrate compared with a high-protein meal, as expected, promoted a greater overall glucose and insulin response. This current study was the first to investigate the effect of macronutrient specific meals on GLP-1 in adolescents. There was no effect of meal type on GLP-1 responses, which is consistent with a study in adults [7], although contrasting others which show protein to be more effective than carbohydrate at stimulating GLP-1 release [6,30]. A point of difference may be protein source and, consequently, differences in their digestion and absorption—for instance, whey protein has been observed to stimulate GLP-1 secretion more than casein [31]. With regard to PYY, protein more so than carbohydrate has previously been shown in both children and adolescents to stimulate its release [12,13].

Although a prolonged suppression of ghrelin after the high-protein meal may have translated to greater satiation, subjective appetite (hunger and fullness) and subsequent energy intake between meals were not different. This is in contrast to the findings by Leidy et al. in a group of adolescents who were healthy to overweight [32]. A point of difference may be food form, in that the meal provided by Leidy et al. was solid compared to the liquid meal in this study. Previous research in adolescents has shown weaker appetitive responses to liquids compared to solid meals [33]. Some reasons may include a faster transit for liquids compared to solids, resulting in a different time course of nutrient exposure to nutrient sensors in the gut; and that the act of chewing of solids could provide a satiety signal not triggered by swallowing liquids [34]. Moreover, it can be suggested that mismatches between appetite hormonal profiles and subjective appetite indicate that the regulation of appetite is complex, involving an interplay of factors other than appetite hormones per se. These include protein metabolites, plasma amino acids, and other physiological and non-physiological cues (e.g., gastric distension and environment, respectively) [7].

There are some limitations that should be considered. Firstly, the sample size in this study was small, which may have confounded the interpretation of some nonsignificant findings. Secondly, total ghrelin was measured, which may not reflect the biologically active form; however, similar changes following meal intake have been documented between the total and active forms [12,35]. A strength of this study was that participants were provided meals tailored to 25% of their estimated energy requirements rather than provided the same caloric meals. In addition, physical activity was controlled for 24 h prior to each study day, which is important since acute exercise can have an influence on postprandial appetite hormonal responses in adolescents [36].

The present study found that adolescents with obesity have a different ghrelin profile compared to those of healthy weight in the fasted state and after eating. The relative suppression of ghrelin (i.e., change from fasting levels) after eating, however, was not different between the two weight status groups. Although the meal high in protein was shown to prolong the suppression of ghrelin response (compared with the high-carbohydrate meal) in adolescents of healthy weight and with obesity, a concomitant subjective feeling of fullness and reduction in energy intake was not observed. To further our understanding of the drivers of food intake and help us comprehend this mismatch, consideration should be given to other physiological factors and environmental cues.

## Figures and Tables

**Table 1:** Baseline characteristics.

	OB (*n* = 8)	HW (*n* = 12)	*p*-value
Age years (range)	16.1 ±0.4 (11.0–17.5)	16.0 ±0.6 (11.0–19.0)	0.921
Male/female, *n*	4/4	4/8	0.648 ^b^
Tanner stage ^a^	4.0 ± 2.0	4.0 ± 4.0	0.642 ^b^
Weight (kg)	98.3 ± 7.9	55.7 ± 2.7	0.001
Weight z-score	2.1 ± 0.2	0.1 ± 0.2	<0.001
Height (cm)	169.0 ± 2.2	164.6 ± 2.3	0.213
BMI (kg/m^2^)	34.4 ± 2.6	20.5 ± 0.8	0.001
BMI z-score	2.1 ± 0.1	0 ± 0.2	<0.001
Waist (cm)	106.7 ± 5.9	73.2 ± 1.5	0.001
Fat mass (kg)	37.3 ± 7.7	13.9 ± 1.8	0.001
% body fat	36.3 ± 4.8	24.3 ± 2.3	0.023
Insulin (µU/mL) ^c^	34.0 ± 24.0	13.7± 5.2	0.008
Glucose (mmol/L)	4.7 ± 0.1	4.6 ± 0.1	0.663
HOMA	5.9 ± 0.9	2.7 ± 0.2	0.012
Ghrelin (pg/mL)	248.0 ± 41.6	546.1 ± 65.0	0.001
GLP-1 (pmol/L)	3.4 ± 1.1	3.5 ± 0.6	0.934
Leptin (pg/mL)	26.7 ± 5.2	6.2 ± 1.3	0.001
PYY (pg/mL) ^d^	98.9 ± 13.9	95.4 ± 20.5	0.891

OB: Obese; HW: Healthy weight; BMI: Body mass index; HOMA: Homeostasis model assessment index; GLP-1: Glucagon like peptide one; PYY: Peptide YY; ^a^ data presented as median (range); ^b^ Fisher’s exact test; ^c^ data expressed as median (interquartile range) for non-normally distributed data; ^d^ obese (*n* = 5) and healthy weight (*n* = 5). Data expressed as mean (SEM).

**Table 2:** The iAUC for ghrelin, glucose, insulin, GLP-1, and PYY in adolescents after meal intake.

	High Protein		High Carbohydrate		*p*-Value Wt	*p*-Value Meal	*p*-Value Wt × Meal
	OB (*n* = 8)	HW (*n* = 12)	OB (*n* = 8)	HW(*n* = 12)			
Ghrelin (pg/mL/4h)	−23361 ± 3919	−64135 ± 9539	−28432 ± 6909	−57592 ± 8984	0.008	0.851	0.149
Glucose ^b^ (mmol/L/4h)	52 ± 141	15 ± 24	342 ± 421	212 ± 233	0.020	<0.001	0.019
Insulin (µU/mL/4h)	30726 ± 6230	11556 ± 1417	42384 ± 12274	14732 ± 1816	0.005	0.046 ^a^	0.236
GLP-1 ^b^ (pmol/L/4h)	911 ± 458	750 ± 380	638 ± 1233	819 ± 544	0.943	0.719	0.837
PYY ^c^ (pg/mL/4h)	14861 ± 6415	6719 ± 2127	6735 ± 2409	8354 ± 1920	0.488	0.354	0.177

OB: Obese; HW: Healthy weight; Wt: Weight; HP: High protein; HCHO: High carbohydrate; GLP-1: Glucagon like peptide one; PYY: Peptide YY; ^a^ not significant when adjusted for gender and pubertal status; ^b^ data expressed as median (interquartile range) for non-normally distributed data; ^c^ obese (*n* = 5) and healthy weight (*n* = 5). Data analysed using a mixed between–within-subjects analysis of variance. Data expressed as mean (SEM).

**Table 3:** Subsequent energy intake in adolescents after high-protein and high-carbohydrate meals.

	High Protein		High Carbohydrate		*p*-Value Wt	*p*-Value Meal	*p*-Value Wt × Meal
	OB (n = 8)	HW (*n* = 12)	OB (*n* = 8)	HW(*n* = 12)			
Lunch (kJ)	3982 ± 402	3205 ± 299	3918 ± 427	3362 ± 265	0.128	0.851	0.653
24 h food record (kJ)	7288 ± 2062	6110 ± 538	7403 ± 1004	6271 ± 656	0.262	0.903	0.984

OB: Obese; HW: Healthy weight; Wt: Weight. Data presented as median (interquartile range) for non-normally distributed data.
